# Does joint-sparing tumor resection jeopardize oncologic and functional outcomes in non-metastatic high-grade osteosarcoma around the knee?

**DOI:** 10.1186/s12957-023-03045-2

**Published:** 2023-06-21

**Authors:** Mengquan Huang, Ziyang Ma, Jie Yu, Yajie Lu, Guojing Chen, Jian Fan, Minghui Li, Chuanlei Ji, Xin Xiao, Jing Li

**Affiliations:** 1 Department of Orthopedics, Xi Jing Hospital, Air Force Medical University, Shaanxi 710032 Xi’an, People’s Republic of China; 2 Department of Orthopedics, 986 Hospital, Air Force Medical University, Shaanxi 710032 Xi’an, People’s Republic of China

**Keywords:** Osteosarcoma, Joint-sparing tumor resection, Ablation, Local recurrence, Surgical margin

## Abstract

**Background:**

We previously reported joint-sparing tumor resection for osteosarcoma with epiphyseal involvement in which transepiphyseal osteotomy went through the in situ ablated epiphysis. However, we do not know whether this is a safe approach when compared with joint-sacrificed tumor resection. Our objective was to compare oncologic and functional outcomes between patients who underwent joint preservation (JP) and joint replacement (JR) tumor resection. Furthermore, we identified the risk factors of local recurrence, metastasis and survival.

**Methods:**

Eighty-nine patients with non-metastatic high-grade osteosarcoma around the knee were treated with limb-salvage surgery (JP in 47 and JR in 42). Age, gender, tumor location, pathologic fracture, plain radiographic pattern, limb diameter change, perivascular space alteration, surgical margin, local recurrence, metastasis, death, and the Musculoskeletal Tumor Society (MSTS)-93 scores were extracted from the records. Univariate analysis was performed to compare oncologic and functional outcomes. Binary logistic and cox regression models were used to identify predicted factors for local recurrence, metastasis, and survival.

**Results:**

Local recurrence, metastasis and overall survival were similar in the JP and JR group (*p* = 0.3; *p* = 0.211; *p* = 0.143). Major complications and limb survival were also similar in the JR and JP group (*p* = 0.14; *p* = 0.181). The MSTS score of 27.06 ± 1.77 in the JP group was higher than that of 25.88 ± 1.79 in the JR group (*p* = 0.005). The marginal margin of soft tissue compared with a wide margin was the only independent predictor of local recurrence (*p* = 0.006). Limb diameter increase and perivascular fat plane disappearance during neoadjuvant chemotherapy were independent predictors for metastasis (*p* = 0.002; *p* = 0.000) and worse survival (*p* = 0.000; *p* = 0.001).

**Conclusions:**

Joint-sparing tumor resection with the ablative bone margin offers advantage of native joint preservation with favorable functional outcomes while not jeopardizing oncologic outcomes compared with joint-sacrificed tumor resection. Surgeon should strive to obtain adequate soft tissue surgical margin decreasing risk of local recurrence. Novel drug regimens might be reasonable options for patients with obvious limb diameter increase and perivascular fat disappearance during chemotherapy.

## Introduction

Histologic studies of resected specimens indicated that the prevalence of epiphyseal involvement of the osteosarcomas is around 81% [1, 2]. It is generally believed that intraarticular resection provides tumor-free margin at the joint end and reduces the risk of local recurrence for the treatment of juxta-articular osteosarcoma [3]. Currently, joint-sparing surgery has been only reserved for patients with osteosarcoma not invading the epiphysis [4]. It means that only 20% of osteosarcomas are eligible for joint-sparing tumor resection [5]. The advantages of joint-sparing tumor resection are preservation of the growth capacity of other end of joint, retention of subchondral bone and ligament with no disturbing the inherent stability and congruence of native joint and the avoidance of complications seen with endoprosthetic reconstruction or osteoarticular allograft replacement following intraarticular tumor resection [6–10].

In attempt to save the native knee for patients with epiphyseal involvement, several authors reported the MRI-guided navigation assisted surgery maximizing the epiphysis preservation with safe bone margins [11–13]. However, this procedure depended largely on the tumor extent and native joint could not be saved if majority of the epiphysis was invaded by tumor. We previously attempted epiphysis-sparing tumor resection for tumor with epiphyseal involvement [14–16]. The core of this procedure was transepiphyseal osteotomy going through the in situ cryo-ablated or microwave ablated tumor-bearing epiphysis, for that previous studies have shown that tumor ablation could release tumor antigens and danger-associated molecular patterns to stimulate T cell immunity, resulting in improved antitumor immunity [17]. Our preliminary study showed no local recurrence in the residual epiphysis and no difference in the occurrence of local recurrence between patients with epiphyseal tumor and metaphysis tumor [16]. However, whether in situ ablation assisted joint-sparing tumor resection jeopardizes the whole oncologic outcomes is still unknown. In addition, it remains unclear whether functional outcomes and complications differ between patients who had their native joint preserved and resected.

The aim of this retrospective study was to determine whether in situ ablation increase the chance of native joint preservation and to compare the oncologic and functional outcomes between patients who had their joint preserved and who had their joint resected in the treatment of non-metastatic osteosarcoma around the knee. Meanwhile, we analyzed initial clinicopathologic characteristics, chemotherapy response and surgical procedure to identify risk factors associated with local recurrence, metastasis and overall survival.

## Patients and methods

### Patient selection

Between 2010 and 2018, we treated 109 patients with non-metastatic high-grade osteosarcomas around the knee at our institution. We retrospectively reviewed 89 cases according to the following criteria: (1) no history of previous treatment except biopsy; (2) both chemotherapy and limb-salvage surgery performed at our institute; (3) with favorable response to chemotherapy. We excluded 7 patients who had amputation or rotationplasty, 2 patients whose treatment did not include neoadjuvant or post-surgery chemotherapy, 2 patients who developed obvious lung metastasis after neoadjuvant chemotherapy and 9 patients lost to follow-up. 49 male (55%) and 40 female (45%) patients with a median age of 13.3 years (range, 7–24 years) were included in this study. 56 (63%) tumors were located at distal femur and 33 (37%) at proximal tibia. Median follow-up of cases was 68.5 months (range, 16 to 162 months). This study was performed in accordance with the Helsinki Declaration. Informed consent was obtained from all patients before study inclusion.

### Management and the definition of study variables

**Fig. 1:**
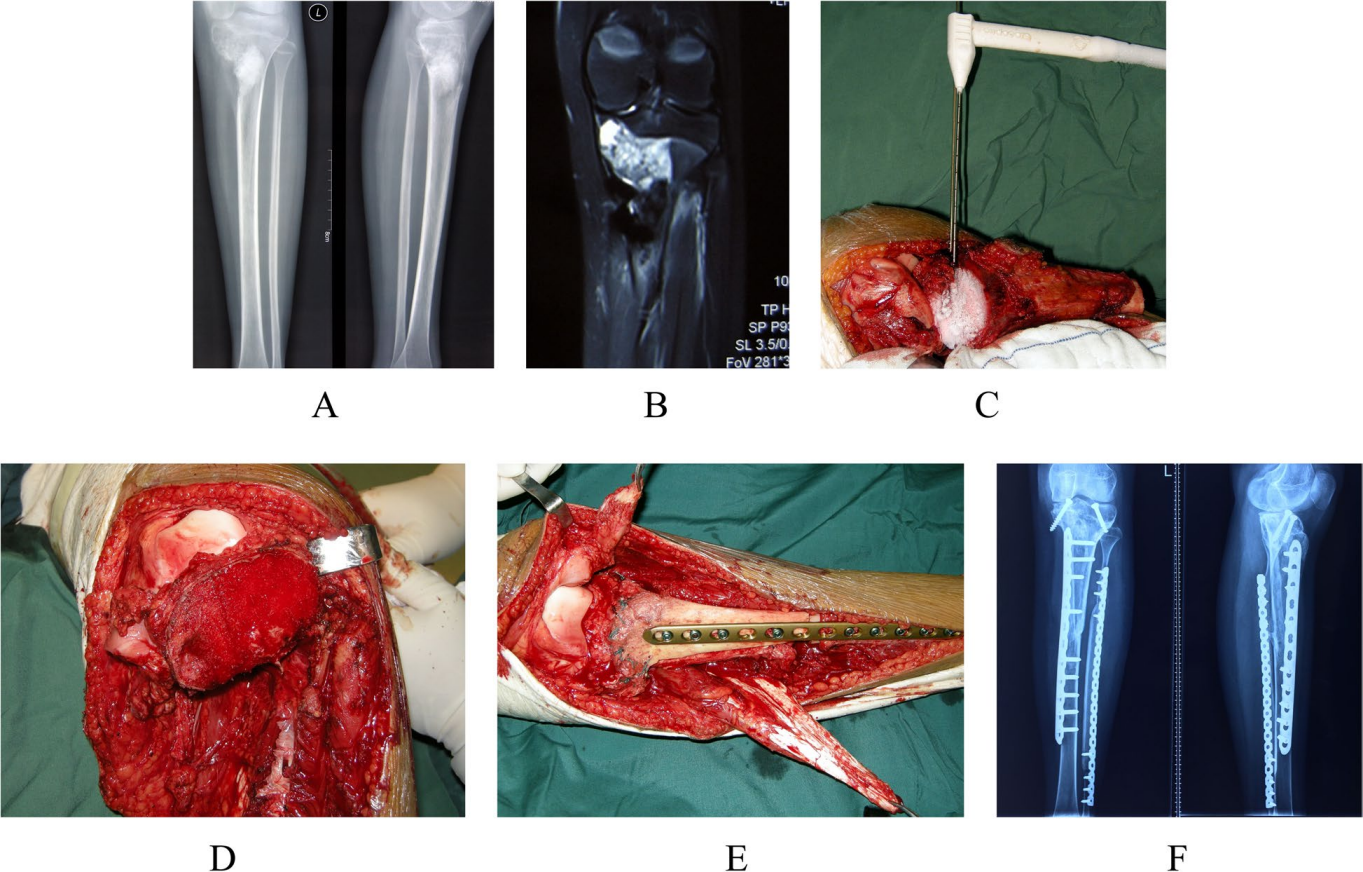
**A**–**F** A thirteen-year-old girl with an osteosarcoma in the proximal aspect of the tibia. **A** Anteroposterior radiograph showing tumor located at the proximal tibia. **B** Coronal magnetic resonance image showing the extent of tumor extension with epiphysis involvement. **C** An intraoperative view showing frosted medial plateau during in situ cryoablation. **D** An intraoperative view showing the preserved epiphysis after transepiphyseal osteotomy. The ablative bone margin was achieved in this case. **E** An intraoperative view showing intercalary reconstruction with recycled tumor-bearing bone and vascularized fibular graft. **F** Anteroposterior radiograph postoperative 54 months showing osteotomies healing with mature callus and mild degenerative change in the medial compartment of the knee. This patient had the MSTS score of 29

All patients underwent neoadjuvant chemotherapy of DIA protocol [18], limb-sparing surgery and postoperative chemotherapy. Definitive limb-sparing surgery was performed within 4 weeks of the completion of neoadjuvant chemotherapy. 47 patients (57%) underwent limb salvage surgery with native joint preserved (JP group) and 42 patients (43%) underwent limb salvage surgery with native joint resected (JR group). A comparison of two treatment groups’ main characteristics identified no significant difference (Table 1). Of the 47 patients in the JP group, 28 patients with epiphyseal involvement underwent transepiphyseal intercalary tumor resection immediately following in situ microwave or argon-based cryoablation of epiphysis [14, 15] (Fig. 1). Nineteen patients who had metaphysis tumor not crossing the growth plate/epiphyseal line underwent tumor resection under the guidance of X-ray fluoroscopy. Intercalary reconstruction in the JP group included allograft in 11 patients, combination of an allograft/devitalized tumor-bearing bone and a vascularized fibula flap in 36. In the JR group, all patients underwent intraarticular tumor resection and reconstruction including osteoarticular allograft in 8 patients, endoprosthesis in 29 and custom-made bi-polar hinge prosthesis in 5.

**Table 1:** Patients’ characteristics of the two treatment groups

Variable	JP	JR	*p* value
Histology			
conventional osteosarcoma	47	42	1.000
Age			
≤ 15 years	31	22	0.193
> 15 years	16	20	
Gender			
Male	24	25	0.625
Female	23	17	
Anatomic location			
Femur	30	26	0.851
Tibia	17	16	
Pathologic fracture			
No	45	40	0.908
Yes	2	2	
Initial tumor volume			
≥ 50 cm^3^	26	30	0.116
< 50 cm^3^	21	12

The factors assessed in the present study were: age, gender, primary tumor location, pathologic fracture, plain radiographic pattern (radiodense, radiolucent and mixed), limb diameter change during chemotherapy, perivascular tissue change on MRI after chemotherapy, surgical margin, local recurrence, distant metastasis, survival, reconstructive complications and the musculoskeletal tumor society (MSTS) scores [19]. Limb diameter change was measured by tape and classified as “increased” (more than 10% increase of maximum original cross-section diameter) or “stable” (increase less than 10%). Specifically, perivascular space change was classified as “stable” (no change of spatial space between major vessels and tumor after chemotherapy on MRI) or “progressed” (intact perivascular fat plane at diagnosis and fat plane disappearance after chemotherapy on MRI). Bone and soft tissue margins were checked using pathologic specimens. Wide margin for bone was defined as a tumor free margin of ≥ 2 cm and limited wide margin for bone was defined as a tumor-free margin of < 2 cm. For the 28 patients who had tumor resection through the in situ ablated tumor-bearing bone, when the residual margin pathology was negative, it reached a sufficient ablative margin [20, 21]. Soft tissue margin was determined according to Enneking definition as “wide”, “marginal” [22]. There was no radical or intralesional margin in this series. Local recurrence was screened according to symptomatology or plain radiographic or bone scan. The metastasis was determined by routine plain radiography, chest CT images, and bone scan. All local and distant relapses were confirmed by histology.

### Statistics

Summary data for normally distributed variables are presented as the mean and standard deviation (SD). Categorical variables are presented as the number of subjects and the percentage of the specified group. Descriptive statistics and univariate methods were used to examine the proportion of patients presenting with local recurrence and distant metastasis according to variables at diagnosis and treatment related factors. The variables that presumably had influenced local recurrence and distant metastasis were entered into binary logistic regression analysis to predict their effect on local recurrence and distant metastasis. The variables in this model were selected for inclusion for univariate values of *p* < 0.1.

Overall survival rate was estimated with use of the Kaplan–Meier method. The log-rank test was used to identify survival differences between the JP and the JR groups. Cox proportional hazards regression models were used to evaluate associations between outcomes of overall survival and related variables. Backward selection was used to identify the variables for the final multiple cox-regression models, which reported results of all factors that remained significant at the 5% level. Fisher’s exact chi-square test was used to identify differences in the occurrence of major complications, survival of the primary reconstruction and survival of limb between the JP and the JR groups.

## Results

### Local recurrence

Local recurrence overall was 11.2% (10 of 89) at final follow-up. The percentage of local recurrence was similar in the JR and JP group (*p* = 0.3; 6 of 42 [14.3%] versus 4 of 47 [8.5%], respectively). However, in the JP group with epiphysis involvement, the rate of local recurrence in the patients without achieved ablative margins was higher than that in the patients with ablative margins (*p* = 0.014; 3 of 7 [43%] versus 1 of 21 [5%], respectively) (Table 2). Nine local recurrences occurred in the soft tissue and one in the bone. Local recurrence is the first sign of recurrent disease in 5 patients, whereas it follows or presents synchronously with distant metastases in 5 patients. Of the 2 patients who had local recurrence without metastasis, one was treated with amputation because of contact with neurovascular structures and the other was treated with resection of the relapsed tumor. Both patients were no evidence of disease at the final follow-up. Of the 3 patients who had local recurrence ahead of lung metastasis, 2 patients underwent amputation and one underwent resection of relapsed tumor. All 3 patients died of metastatic disease.

**Table 2:** Local recurrence in JP group with epiphysis involvement

Variable	Yes	No	*p* value
Bony resection margin			
Ablative margin	1	20	0.014
Without ablative margin	3	4	

We found that age, gender, primary tumor location, plain radiographic pattern, epiphysis involvement and bone margin did not influence the local recurrence. Limb diameter increase and presence of pathologic fracture tend to lead to local recurrence (*p* = 0.058, 0.061, respectively). Patients with marginal margin in soft tissue were related to local recurrence as compared with those with wide margin (*p* = 0.011). There were no differences in local recurrence among patients with wide, limited wide and ablative bone margin (*p* = 0.496). After controlling for relevant confounding variables, the logistic model revealed that marginal margin in soft tissue, (*p* = 0.006; relative risk [RR], 8.982; 95% confidence interval [CI], 1.896–43.171) was only independent risk predictor of local recurrence (Table 3).

**Table 3:** Univariate and binary logistic models for local recurrence and metastasis

	Univariate models			Multivariate binary logistic model			Univariate models			Multivariate binary logistic model		
Variable	Local recurrence		*p* value	OR	95% CI	*p* value	Metastasis		*p* value	OR	95% CI	*p* value
	Yes	No					Yes	No				
Age												
≤ 15	8 (80%)	45 (56.9%)					17 (68%)	36 (56.3%)				
> 15	2 (20%)	34 (43.1%)	0.193				8 (32%)	28 (43.7)	0.346			
Gender												
Male	7 (70%)	42 (53.2%)					16 (64%)	33 (51.6%)				
Female	3 (30%)	37 (46.8%)	0.254				9 (36%)	31 (48.4%)	0.206			
Anatomic location												
Femur	5 (50%)	51 (64.6%)					17 (68%)	39 (60.9%)				
Tibia	5 (50%)	28 (35.4%)	0.286				8 (32%)	25 (39.1%)	0.357			
Plain radiographic pattern												
Blastic	4 (40%)	37 (46.8%)					9 (36%)	32 (50%)				
Lytic or mixed	6 (60%)	42 (53.2%)	0.474				16 (64%)	32 (50%)	0.17			
Epiphysis involvement												
No involvement	1 (10%)	18 (22.8%)					2 (8%)	17 (26.6%)				
Involvement	9 (90%)	61 (77.2%)	0.321				23 (92%)	47 (73.4%)	0.045			
Pathologic fracture												
No	8 (80%)	77 (97.5%)					24 (96%)	61 (95.3%)				
Yes	2 (20%)	2 (2.5%)	0.061				1 (4%)	3 (4.7%)	0.687			
Perivascular space change												
Stable	9 (90%)	58 (73.4%)					8 (32%)	59 (92.2%)				
Progressed	1 (10%)	21 (26.6%)	0.234				17 (68%)	5 (7.8%)	< 0.001	93.863	10.537–836.106	< 0.001
Limb diameter increase												
Stable	4 (40%)	56 (70.9%)					9 (36%)	51 (79.7%)	< 0.001			
Increased	6 (60%)	23 (29.1%)	0.058				16 (64%)	13 (20.3%)		31.016	3.703–259.793	0.002
Bony resection margin												
Wide	7 (70%)	44 (55.7%)					16 (64%)	35 (54.7%)				
Limited wide	2 (20%)	15 (19%)					4 (16%)	13 (20.3%)				
Ablated	1 (10%)	20 (25.3%)	0.496				5 (20%)	16 (25%)	0.725			
Soft tissue margin												
Wide	5 (50%)	69 (87.3%)					19 (76%)	55 (85.9%)				
Marginal	5 (50%)	10 (12.7%)	0.011	8.982	1.869–43.171	0.006	6 (24%)	9 (14.1%)	0.206			
Native joint status												
Preservation	4 (40%)	43 (54.4%)					11 (44%)	36 (56.2%)				
Resection	6 (60%)	36 (45.6%)	0.3				14 (56%)	28 (43.8%)	0.211			

### Metastatic disease

The metastatic disease rate overall was 28.1% (25 of 89). Primary metastases were located in the lung in 22 patients and in both the lung and the bone in 3 patients. Of the 5 patients who had pulmonary lobectomy and 1 who had radiofrequency ablation, 2 had no evidence of disease, 1 patient was alive with disease and 3 died of the disease.

With the numbers available, factors such as age, gender, primary tumor location, plain radiographic pattern, bone margin, soft tissue margin, and JR/JP condition were similar for developing metastatic disease. Epiphyseal tumor involvements, disappearance of the perivascular fat during chemotherapy and limb diameter increase were related to metastasis (*p* = 0.045, 0.000, 0.000, respectively) in the univariate analysis. After adjusting for confounding variables, limb diameter increase (*p* = 0.002; RR, 31.016; 95% CI, 3.703–259.793) and disappearance of the perivascular fat during chemotherapy (*p* = 0.000; RR, 93.863; 95% CI, 10.537–836.106) were found to be independent negative predictors for distant metastasis (Table 3).

### Overall survival

**Fig. 2:**
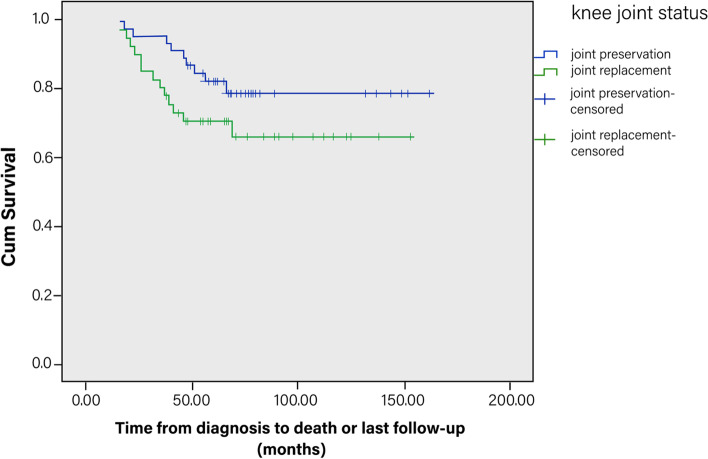
The Kaplan–Meier overall survivorship (end point was date of death or date of last follow-up) showing similar survival of the joint preservation group versus the joint replacement group: 81% versus 72% at 5 years and 79% versus 63% at 10 years, respectively

Twenty-two patients died of pulmonary metastases. The overall Kaplan–Meier survival was 78.1% at 5 years and 76.2% at 10 years. Survivorship was also similar (*p* = 0.143) with survival of the JP group versus the JR group being 81% versus 72% at 5 years and 79% versus 63% at 10 years, respectively) (Fig. 2). Limb diameter increase greater than 10% (HR, 8.886; 95% CI, 2.291–27.034; *p* = 0.000) and perivascular fat plane disappearance during chemotherapy (HR, 4.820; 95% CI, 1.920–12.097; *p* = 0.001) were independent poor prognostic factors for patient survival and no other independent predictors of survival were identified (Table 4).

**Table 4:** Risk factors for overall survival

Variable	Survival		*p* value	HR	95% CI	*p* value from cox regression
	Survival	Death				
Perivascular space change						
No change	59 (88.1%)	8 (11.9%)		1		
Disappeared	8 (36.4%)	14 (63.6%)	< 0.001	4.820	1.920–12.097	0.001
Limb diameter change						
Stable	54 (90%)	6 (10%)		1		
Increased	13 (43.3%)	16 (56.7%)	< 0.001	8.886	2.291–27.034	< 0.001

### Major complications and functional outcomes

Of the 89 patients in the study, 53 (59%) achieved healing without reconstructive or oncologic complications. Additional surgical procedures were performed including 9 to treat oncologic complications and 30 to treat reconstructive complications. In the JP group, 4 patients with nonunion, of which 1 had primary reconstruction removal and 3 had bone graft. Two patients with fracture removed primary reconstruction. Three patients with wound problems were treated with de´bridement and rotational full-thickness skin. Three patients with deep infection, of which 1 had primary reconstruction removal, 1 underwent amputation and 1 had subcutaneous flap transplantation. Three patients with local recurrence, amputation in 2 and resection and reconstruction in 1. Two patients with distant metastasis were treated with pulmonary lobectomy. In the JR group, 1 patient with fracture was treated with internal fixation. Two patients with wound problems were treated with de´bridement and rotational full-thickness skin. Five patients with deep infection, 5 patients with aseptic loosening and 3 patients with prosthesis breakage, they all removed primary reconstruction. Two patients with polyethylene wear, of which 1 had primary reconstruction removal and 1 had arthroscopic therapy. Two patients with local recurrence underwent amputation. Two patients with distant metastasis were treated with pulmonary lobectomy (Tables 5 and 6).

**Table 5:** Major complications need additional surgery

Group	Orthopaedic							Oncologic			
	Nonunion	Fracture	Wound problems	Deep infection	Prosthesis breakage	Aseptic loosening	Polyethylene wear	Local recurrence	Distant metastasis	Total	*p* value
JP (47)	4 (23.5%)	2 (11.8%)	3 (17.6%)	3 (17.6%)				3 (17.6%)	2 (11.8%)	17(100%)	0.14
JR (42)		1 (4.5%)	2 (9.1%)	5 (22.7%)	3 (13.6%)	5 (22.7%)	2 (9.1%)	2 (9.1%)	2 (9.1%)	22 (100%)

**Table 6:** Reasons for primary reconstruction removal and amputation

Group	Reasons for primary reconstruction removal								Reasons for amputation				
	Nonunion	Fracture	Deep infection	Prosthesis breakage	Aseptic loosening	Polyethylene wear	Total number		*p* value	Local recurrence	Deep infection	Total number	*p* value
JP (47)	1 (25%)	2 (50%)	1 (25%)				4 (100%)		0.007	2 (66.7%)	1 (33.3%)	3 (100%)	0.181
JR (42)			5 (35.7%)	3 (21.4%)	5 (35.7%)	1 (7.2%)	14 (100%)			2 (28.6%)	5 (71.4%)	7 (100%)	

Major complications and amputation were similar in the JP and JR group (17/47 versus 22/42, *p* = 0.14; 3/47 versus 7/42, *p* = 0.181) (Tables 5 and 6). Original reconstructions of the JP group were more likely to survive than those of the JR group (4/47 versus 14/42, *p* = 0.007) (Table 6). Forty-four patients in the JP group and 34 patients in the JR group were available for final functional evaluation. The MSTS score of 27.06 ± 1.77 in the JP group was higher than that of 25.88 ± 1.79 in the JR group (*p* = 0.005).

## Discussion

We observed no difference in local recurrence and distant metastasis between the JR group and the JP group. Meanwhile, overall patient survival was also similar in both groups. These comparative outcomes suggested that joint-preserved limb sparing surgery with the aid of adjuvant co-treatment is not detrimental to local and systemic oncologic control when compared with joint-sacrificed limb sparing surgery. The major complications were similar in both groups. Given the high MSTS score and limb survival in the JP group, retaining native joint is preferable, particularly in children and adolescents whose growth plates are open. Furthermore, we identified marginal surgical margin of soft tissue was an independent risk factor in predicting local recurrence and found that limb diameter increased and disappearance of perivascular fat plane during neoadjuvant chemotherapy predict distant metastasis and poor overall survival.

There were limitations to our study. First, it was a retrospective study not allowing us to capture some important information but only what was listed in the medical records. For instance, we did not evaluate some known prognostic factors such as tumor necrosis rate due to its unavailability for some patients who underwent reconstruction with recycled tumor-bearing bone. Regarding histologic response to preoperative chemotherapy, tumor necrosis rate remains the most powerful treatment related prognostic factors [23]. However, it is generally available a week after surgery and preoperative evaluation thus depends upon clinical and radiographic assessment. Therefore, we introduced clinical and image evaluation as complement assessing chemotherapy response. The reason why we utilized MRI study observing perivascular space is that the intact fat will be a final barrier before tumor sheathing on the vascular side. Tumor enlargement has been regarded as a predictor for poor prognosis [24]; therefore, limb diameter increase was a potential prognostic factor similar to tumor volume change. However, defining limb diameter increase using a cutoff of 10% as indictor of volume enlargement was sort of exploratory.

The second limitation to our study was introducing the concept of ablative margin. In attempt to preserve the joint with tumor involving epiphysis, we resected tumor through in situ-ablated tumor-bearing bone. There was no described margin for this surgical resection previously. Although pathologic examination of specimen revealed no live tumor at the osteotomy site, we could not obtain direct histologic evidence of no live tumor in the residual epiphysis [14]. Therefore, we admit the concept of ablative margin is path breaking attempt in nature due to the utilization of the in situ ablation technique and we hope that future researches will verify the efficacy of this surgical procedure.

Surgical margin has been a fundamental issue related to local recurrence [25]. In order to differentiate the influence of the different margin status on the safety of joint preservation, we separated the axial margin and longitudinal margin in this study. Axial margin is mainly relevant to the soft tissue. We found that marginal margin in the soft tissue was a risk prognostic factor for local recurrence in multivariate analysis, which was in agreement with previous studies [26]. However, whether joint end could be safely preserved is mostly determined by the extent of longitudinal resection margin of the bone. The crucial issue is how close we can approach tumor to save the nearby joint end safely. Most surgeons believed that epiphysis preservation should only be reserved for tumor at least 2 cm away from the articular surface or tumor not invading the epiphysis [27]. Previous studies have revealed that the prevalence of transepiphyseal spread of the tumor is around 81% [1, 2], suggesting only approximate 20% of osteosarcomas are eligible for joint preservation surgery, which have been verified in early limb-salvage reports [5]. In the current series, the prevalence of tumor invading epiphysis is 78.6%, which is similar to previously reported rates [1, 2, 5]. Differently, almost half of these patients with epiphyseal tumor involvement had their native joint preserved. This is mainly due to tumor resection via the ablated tumor-bearing bone. It subsequently raises the query: whether ablated surgical margin would lead to increase of local recurrence? We found there was no statistical difference in the effect of the wide and ablated margin on local recurrence. Therefore, ablated bone margin is adequate and comparable to wide bone margin in terms of its influence on local relapse.

Our second question was to determine prognostic factors for metastasis and Patient’s survival. In multivariate analysis, we found that limb diameter increased and perivascular fat plane disappearance during neoadjuvant chemotherapy were independent predictors of metastasis and poor survival. Regarding tumor volume change, it has received little attention as a viable prognostic factor because usually there is not a marked volumetric response during neoadjuvant chemotherapy [27]. Recently, some report suggested reduced or stable tumor size cannot guarantee a good response, increase in tumor volume is well correlated with a poor histologic response [28, 29]. Therefore, this factor is regarded as one of the parameters that may predict poor histologic response to preoperative chemotherapy [30]. Our results are consistent with previous report in which the authors found that tumor enlargement after chemotherapy has a greater relative effect on survival than any other factor, including initial tumor size, surgical margin, and histologic response [31].

Conceptually, distant metastasis could be dependent on direct vascular or lymphatic pathways. The major vessels invasion would be a main culprit for subsequent metastasis and worse survival [32]. MRI can reveal gross encasement of a vessel readily, but it usually cannot differentiate mere contact, adherence or subtle invasion if no tissue plane is evident between the tumor and the vessels [33]. We are not fully certain whether tumor closely abutting major vessel represent possible metastatic path or seeding mechanism, however, gradual perivascular fat disappearance during chemotherapy was an independent prognostic factor in predicting metastasis and worse survival in the current study. Therefore, we suspect the disappearance of perivascular fat during chemotherapy may lead to progressive vessel or lymphatic invasions and subsequent tumor seeding by circulating tumor cells. However, we did not have histopathologic proof of this correlation and it should be under further investigation in the future.

## Conclusion

Tumor resection with the ablative bone margin increases the chance of joint preservation. The joint preservation offers relative better functional outcomes while incurring similar risk of local recurrence and metastasis as those offered by joint sacrificing tumor resection. Inadequate soft tissue margin is a powerful prognostic factor for local recurrence and surgeon should strive to obtain adequate margins. Two easily assessable clinical factors (progressive disappearance of perivascular fat plane and limb diameter increase during preoperative chemotherapy) are associated with an increased metastasis and worse survival in patients with localized osteosarcomas. Given that current efforts to treat these refractory groups are ineffective; these risk factors should be considered when deciding risk-adapted treatments such as a clinical trial or novel drug regimens for osteosarcoma patients.

## Data Availability

The data that support the findings of this study are available on request from the corresponding authors (Jing Li and Xin Xiao).

## References

[1] Saifuddin A, Sharif B, Gerrand C, Whelan J (2019). The current status of MRI in the pre-operative assessment of intramedullary conventional appendicular osteosarcoma. Skeletal Radiol.

[2] San-Julian M, Aquerreta JD, Benito A, Cañadell J (1999). Indications for epiphyseal preservation in metaphyseal malignant bone tumors of children: relationship between image methods and histological findings. J Pediatr Orthop.

[3] Zimel MN, Cizik AM, Rapp TB, Weisstein JS, Conrad EU (2009). Megaprosthesis versus Condyle-sparing intercalary allograft: distal femoral sarcoma. Clin Orthop Relat Res.

[4] Hamed Kassem Abdelaal A, Yamamoto N, Hayashi K, Takeuchi A, Miwa S, Tsuchiya H (2015). Epiphyseal sparing and recon-struction by frozen bone autograft after malignant bone tumor resection in children. Sarcoma.

[5] Muscolo DL, Ayerza MA, Aponte-Tinao LA, Ranalletta M (2004). Partial epiphyseal preservation and intercalary allograft recon-struction in high-grade metaphyseal osteosarcoma of the knee. J Bone Joint Surg Am.

[6] Tsuchiya H, Abdel-Wanis ME, Sakurakichi K, Yamashiro T, Tomita K (2002). Osteosarcoma around the knee. Intraepiphyseal ex-cision and biological reconstruction with distraction osteogenesis. J Bone Joint Surg Br.

[7] Takeuchi A, Yamamoto N, Hayashi K, Matsubara H, Miwa S, Igarashi K, Tsuchiya H (2019). Joint-preservation surgery for pediatric os-teosarcoma of the knee joint. Cancer Metastasis Rev.

[8] Kiss S, Terebessy T, Szöke G, Kiss J, Antal I, Szendröi M (2013). Epiphysis preserving resection of malignant proximal tibial tumours. Int Orthop.

[9] Manfrini M, Gasbarrini A, Malaguti C, Ceruso M, Innocenti M, Bini S, Capanna R, Campanacci M (1999). Intraepiphyseal resection of the proximal tibia and its impact on lower limb growth. Clin Orthop Relat Res.

[10] Henderson ER, Groundland JS, Pala E, Dennis JA, Wooten R, Cheong D, Windhager R, Kotz RI, Mercuri M, Funovics PT, Hornicek FJ, Temple HT, Ruggieri P, Letson GD (2011). Failure mode classification for tumor endoprostheses: retrospective review of five institutions and a literature review. J Bone Joint Surg Am.

[11] Cho HS, Oh JH, Han I, Kim HS (2009). Joint-preserving limb salvage surgery under navigation guidance. J Surg Oncol.

[12] Kim JH, Kang HG, Kim HS (2010). MRI-guided navigation surgery with temporary implantable bone markers in limb salvage for sarcoma. Clin Orthop Relat Res.

[13] Thompson MJ, Shapton JC, Punt EP, Johnson CN, Conrad EU (2018). MRI identification of the osseous extent of pediatric bone sarcomas. Clin Orthop Relat Res.

[14] Li J, Guo Z, Wang Z, Fan H, Fu J (2015). Does microwave ablation of the tumor edge allow for joint-sparing surgery in patients with osteosarcoma of the proximal tibia?. Clin Orthop Relat Res.

[15] Li J, Guo Z, Yang Q, Ji C, Wang Z (2015). Adjuvant argon-based cryoablation for joint-preserving tumor resection in patients with juxta-articular osteosarcoma around the knee. Cryobiology.

[16] Li J, Wang Z, Ji C, Chen G, Liu D, Zhu H (2017). What are the oncologic and functional outcomes after joint salvage resections for juxtaarticular osteosarcoma about the knee?. Clin Orthop Relat Res.

[17] Hoover AR, Kaabinejadian S, Krawic JR, Sun XH, Naqash AR, Yin Q, et al. Localized ablative immunotherapy drives de novo CD8(+) T-cell responses to poorly immunogenic tumors. J Immunother Cancer. 2022;10(10):e004973. 10.1136/jitc-2022-004973 PMC9577935 36253002

[18] Xu M, Wang Z, Yu XC, Lin JH, Hu YC (2020). Guideline for limb-salvage treatment of osteosarcoma. Orthop Surg.

[19] Enneking WF, Dunham W, Gebhardt MC, Malawar M, Pritchard DJ (1993). A system for the functional evaluation of reconstructive procedures after surgical treatment of tumors of the musculoskeletal system. Clin Orthop Relat Res.

[20] Hasegawa T, Takaki H, Kodama H, Matsuo K, Yamanaka T, Nakatsuka A, Takao M, Gobara H, Hayashi S, Inaba Y, Yamakado K (2023). Impact of the ablative margin on local tumor progression after radiofrequency ablation for lung metastases from colorectal carcinoma: supplementary analysis of a phase II trial (MLCSG-0802). J Vasc Interv Radiol.

[21] Yang Q, Qi H, Zhang R, Wan C, Song Z, Zhang L, Fan W (2017). Risk factors for local progression after percutaneous radiofrequency ablation of lung tumors: evaluation based on a review of 147 tumors. J Vasc Interv Radiol.

[22] Enneking WF, Spanier SS, Goodman MA (2003). A system for the surgical staging of musculoskeletal sarcoma. 1980. Clin Orthop Relat Res.

[23] Bertrand TE, Cruz A, Binitie O, Cheong D, Letson GD (2016). Do surgical margins affect local recurrence and survival in extremity, nonmetastatic, high-grade osteosarcoma?. Clin Orthop Relat Res.

[24] Bacci G, Longhi A, Versari M, Mercuri M, Briccoli A, Picci P (2006). Prognostic factors for osteosarcoma of the extremity treated with neoadjuvant chemotherapy: 15-year experience in 789 patients treated at a single institution. Cancer.

[25] Li X, Moretti VM, Ashana AO, Lackman RD (2012). Impact of close surgical margin on local recurrence and survival in osteosarcoma. Int Orthop.

[26] Picci P, Sangiorgi L, Bahamonde L, Aluigi P, Bibiloni J, Zavatta M, Mercuri M, Briccoli A, Campanacci M (1997). Risk factors for local recurrences after limb-salvage surgery for high-grade osteosarcoma of the extremities. Ann Oncol.

[27] Aponte-Tinao L, Ayerza MA, Muscolo DL, Farfalli GL (2015). Survival, recurrence, and function after epiphyseal preservation and allograft reconstruction in osteosarcoma of the knee. Clin Orthop Relat Res.

[28] He F, Qin L, Bao Q, Zang S, He Q, Qiu S, Shen Y, Zhang W (2019). Pre-Operative chemotherapy response assessed by contrast-enhanced MRI can predict the prognosis of Enneking surgical margins in patients with osteosarcoma. J Orthop Res.

[29] Shin KH, Moon SH, Suh JS, Yang WI (2000). Tumor volume change as a predictor of chemotherapeutic response in osteosarcoma. Clin Orthop Relat Res.

[30] Song WS, Jeon DG, Kong CB, Cho WH, Koh JS, Lee JA, Yoo JY, Jung ST, Shin DS, Lee SY (2011). Tumor volume increase during preoperative chemotherapy as a novel predictor of local recurrence in extremity osteosarcoma. Ann Surg Oncol.

[31] Kim MS, Lee SY, Cho WH, Song WS, Koh JS, Lee JA, Yoo JY, Jung ST, Jeon DG (2009). Effect of increases in tumor volume after neoadjuvant chemotherapy on the outcome of stage II osteosarcoma regardless of histological response. J Orthop Sci.

[32] Kawaguchi N, Ahmed AR, Matsumoto S, Manabe J, Matsushita Y (2004). The concept of curative margin in surgery for bone and soft tissue sarcoma. Clin Orthop Relat Res.

[33] Panicek DM, Hilton S, Schwartz LH (1997). Assessment of neurovascular involvement by malignant musculoskeletal tumors. Sarcoma.

